# A novel approach to improve hamstring flexibility: A single-blinded randomised clinical trial

**DOI:** 10.4102/sajp.v75i1.465

**Published:** 2019-04-23

**Authors:** Faris Alshammari, Eman Alzoghbieh, Mohammad Abu Kabar, Mohannad Hawamdeh

**Affiliations:** 1 Department of Physical and Occupational Therapy, School of Applied Health Sciences, The Hashemite University, Zarqa, Jordan

**Keywords:** hamstring, flexibility, stretch, neurodynamic, quadriceps, activation

## Abstract

**Background:**

The hamstrings play a major role in body posture. Shortening or tightness of the hamstrings affects postural alignment and results in possible musculoskeletal pain.

**Objectives:**

The aim of this study was to develop a novel approach to improve hamstring flexibility in young adults.

**Method:**

A single-blinded randomised clinical trial included 60 participants aged 18–24 with shortened hamstrings recruited from the Hashemite University, Zarqa, Jordan. The range of motion of knee extension was measured with the hip at 90° flexion using a simple goniometer to detect the level of hamstring flexibility. Participants received either a passive hamstring stretch (PS), followed by two sets of 10 tibial nerve neurodynamic technique (ND), or PS followed by three sets of 10 repetitions of active knee extension–quadriceps activation (QA), or PS only.

**Results:**

There was a significant improvement of hamstring flexibility in the QA group compared to the PS group (13.4 ± 12.1° vs. 6.2 ± 6.4°, *p* = 0.05). There was a significant improvement in hamstring flexibility post-intervention compared to pre-intervention in the PS group by 6.2 ± 6.4 (30.5 ± 10.8° vs. 36.6 ± 9.5°, *p* = 0.001), ND group by 9.3 ± 6.2 (26.7 ± 10.9° vs. 36.0 ± 9.5°, *p* = 0.001) and QA group by 13.4 ± 12.1 (20.3 ± 9.0° vs. 33.4 ± 8.9°, *p* = 0.001).

**Conclusion:**

Quadriceps muscle activation following passive stretching of the hamstrings appears to be superior to the PS and ND techniques in improving hamstring muscle flexibility.

**Clinical implications:**

Quadriceps activation following passive hamstring stretching can be used in physiotherapy settings to improve hamstring muscle flexibility.

## Introduction

The hamstrings is major muscles that control the movement of the hip and knee joints (Bregenhof et al. 2018;Malfait et al. 2016;Pinniger, Steele &Groeller 2000) and control the alignment of the pelvis and spine (Jozwiak, Pietrzak &Tobjasz 1997). So they play an important role in postural alignment where the shortening of the hamstrings could result in a posterior pelvic tilt and hypolordosis of the lumbar spine (Borman, Trudelle-Jackson & Smith 2011;Jozwiak et al. 1997).

The changes in body posture resulting from hamstring shortening could result in lower back and lower limb pain including hip, knee or ankle joint pain (Jozwiak et al. 1997;Radwan et al. 2014; Sadler et al. 2017; Sanchez-Zuriaga, Artacho-Perez & Bivia-Roig 2016;Witvrouw et al. 2001). A strong relationship has been shown between limited hamstring flexibility and the incidence of low back pain (Sadler et al. 2017). Similarly there appears to be a significant relationship between limited flexibility of the hamstrings and quadriceps and patellar tendinitis and tendinopathy (Morton et al. 2017;Witvrouw et al. 2001).

The prevalence of hamstring muscle tightness is fairly high (Nishchal Ratna Shakya 2018) and appears to be increasing among the youth as is shown in hamstring tightness among undergraduate physical therapy students in Nepal at 40.17% (Nishchal Ratna Shakya 2018). Because hamstring tightness affects body posture, resulting in musculoskeletal pain, it is important to develop a new effective way to improve hamstring flexibility(Borman et al. 2011;Jozwiak et al. 1997).

Many studies have been conducted on hamstring muscle stretching techniques and flexibility (Cini, De Vasconcelos & Lima 2017; Cipriani et al. 2012; Freitas et al. 2015; Nishikawa et al. 2015).Cini et al. (2017) studied the effects of different periods of passive stretching of the hamstrings on hip and knee joint flexibility by comparing one group that received 30 seconds of passive stretching and a second group that received 60 seconds of passive stretching. There was a significant improvement in knee and hip flexibility within both groups, but there was no significant difference in hip or knee flexibility between the groups (Cini et al. 2017), indicating that a 30 seconds stretch is as effective as a 60 seconds stretch. Cipriani et al. (2012) studied the effect of gender and stretch frequency on hamstring muscle flexibility, finding that stretching of the hamstrings three times per week was as effective as stretching once daily. They also did not find a difference in hamstring flexibility improvement between genders (Cipriani et al. 2012).

A longer duration of hamstring stretching is important in reducing the passive torque of the hamstrings, which results in an improvement of hamstring flexibility (Freitas et al. 2015). Johnson et al. (2014) compared stretches of 10 seconds with 9 repetitions or 30 seconds with three repetitions over a 6-week period. They found that the hamstring flexibility increased with 90 seconds of stretching regardless of the duration of the stretch or the number of repetitions (Johnson et al. 2014).

Nishikawa et al. (2015) found that passive stretching was more effective than active stretching in increasing hamstring flexibility (Nishikawa et al. 2015). Self-myofascial stretching improves hamstring flexibility, and applying ultrasound prior to stretching has no effect on hamstring muscle flexibility(Cho & Kim 2016).

Passive stretching of hamstring muscles improves flexibility. However, the use of nerve gliding or quadriceps muscle activation in conjunction with passive stretching of hamstrings could result in more flexibility. In fact, hamstring flexibility may reduce the risk of musculoskeletal pain. Therefore, it is important to develop more effective ways to improve hamstring flexibility.

The purpose of this study was to develop a new effective approach to improve hamstring flexibility. In this study, a tibial nerve neurodynamic technique (ND) and passive hamstring stretching (PS) were compared with active knee extension–quadriceps activation (QA) and PS.

## Methods

A single-blinded randomised clinical trial included participants assigned randomly into three treatment groups using a blocked design with random distribution. Between April 2017 and July 2017, a total of 60 participants between 18 and 24 years old were recruited from students at the Hashemite University. Participants were recruited using flyers. They were included in this study if they had limited flexibility in their hamstrings, defined as a limitation in knee extension of 20° or more, with 90° hip flexion. Participants were healthy and had no history of low back or lower limb injuries. They were excluded from the study if they had a history of lower back, hip joint or knee joint pathologies.

The first author screened the participants prior to participation in order to assure compliance with the inclusion and exclusion criteria.

### Measurement of hamstring muscle flexibility

Hamstring muscle flexibility was measured through the degree of limitation in the knee extension range of motion (ROM). A double-arm universal goniometer (UG) (Baseline, Albany, NY, USA) was used to measure the knee extension ROM. The UG is a valid and highly reliable tool in measuring knee joint ROM (Brosseau et al. 2001). The inter-tester reliability of the UG is 0.977–0.982 and the intratester reliability is 0.972–0.985. The pre- and post-intervention measures for each participant were taken by the same physiotherapist.

**FIGURE 1:**
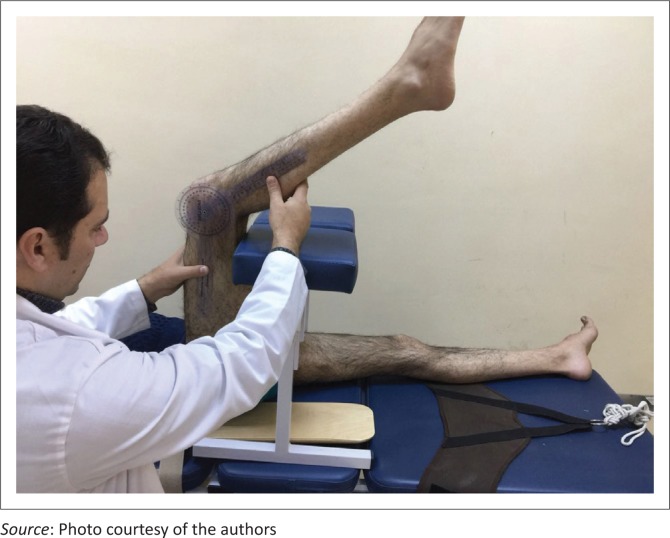
Measuring hamstring muscle flexibility through knee extension range of motion.

Participants were placed in supine, holding their hip joint at 90° flexion (Figure 1). Following that, the participants were asked to extend their knee actively to their maximum ability while keeping the hip joint blocked at 90° flexion. Knee extension ROM was measured at this point to determine the level of hamstring flexibility. The hamstrings were considered to have limited flexibility if the ROM limitation was 20° or more (Feland et al. 2001; Hamid, Ali & Yusof 2013). Assessors were blinded to each participant’s allocation group to avoid any bias towards a specific intervention.

### Intervention

The intervention was conducted by three physiotherapists trained in neurodynamic and quadriceps activation techniques. The intervention included three different techniques as follows:

1. Passive hamstring stretch: Participants were placed in supine while the hip was maintained at 90° flexion. Passive knee extension done by the therapist was applied until the patient reached the maximum level of tolerable stretch. The stretch was sustained for 30 seconds each time. Each patient received three repetitions of passive stretch (Bandy & Irion 1994; Kisner 2012).
2. Tibial nerve neurodynamic technique following PS: Participants received PS. Following that, participants were placed in supine with the hip flexed and the knee extended. Then repetitive ankle dorsiflexion was conducted with eversion in synchronisation with knee flexion–extension in order to apply a sliding–gliding mechanism on the tibial nerve. Two sets of 10 repetitions were applied to glide the tibial nerve through manipulating the ankle and knee positions (Butler 2005).
3. Quadriceps activation following PS: Participants received PS. Following that, participants were asked to extend their knee joint actively while the hip was in 90° flexion. Active knee extension was applied for three sets of 10 repetitions (Kisner 2012).

Following these procedures, hamstring muscle flexibility was measured as was done prior to the intervention.

### Statistical analysis

Data were analysed using the Statistical Package for the Social Sciences version 21. The general characteristics of the participants were summarised using means and standard deviations (SDs) for quantitative variables and frequencies and percentages for categorical variables. A mixed factorial analysis of variance and one-way repeated-measures analysis of variance were used to examine the difference in mean hamstring muscle flexibility (knee ROM) between and within participant groups. Cohen’s *d* was used to determine effect sizes. The level of significance was set at 0.05.

### Ethical considerations

All protocols and procedures were approved by the Institutional Review Board of the Hashemite University (approval number RA/222/1703674). All participants signed a statement of informed consent after the study procedures were explained in detail by the first author.

## Results

The demographics of the participants are shown in Table 1. There were no significant differences in age, height or weight among groups. There was no significant difference in baseline measurements among groups.

**TABLE 1:** Demographic characteristics by study group (*n* = 60).

Variable	Neurodynamic	Quad activation	Passive stretching	*p*
Age (years)	21.5 ± 0.9	21.7 ± 0.8	21.5 ± 0.6	0.657
Weight (kg)	63.7 ± 10.2	62.1 ± 10.9	64.5 ± 10.8	0.752
Height (cm)	166.7 ± 11.9	168.4 ± 12.1	169.8 ± 11.9	0.749

SD, standard deviation.

Data are mean ± SD values.

**FIGURE 2:**
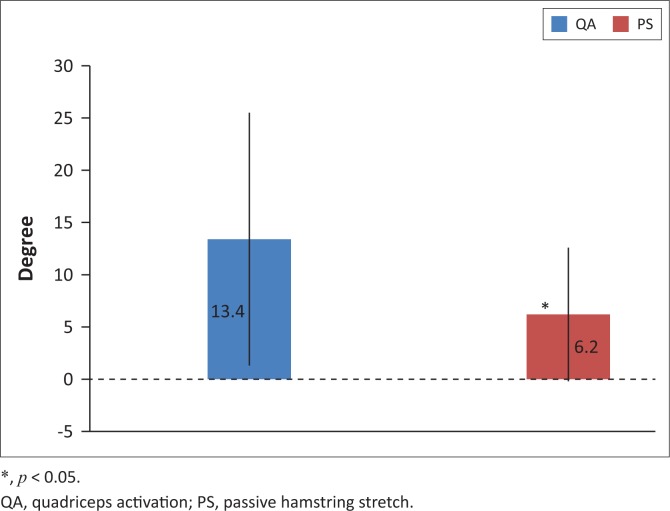
Mean improvement of hamstring muscle flexibility (in degrees) ± standard deviation between quadriceps activation and passive stretch groups.

**FIGURE 3:**
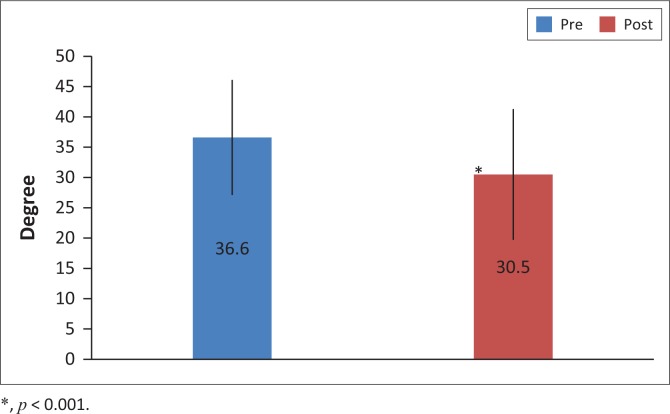
Mean knee extension range of motion (in degrees) ± standard deviation pre- and post-intervention in the group that received passive stretching.

**FIGURE 4:**
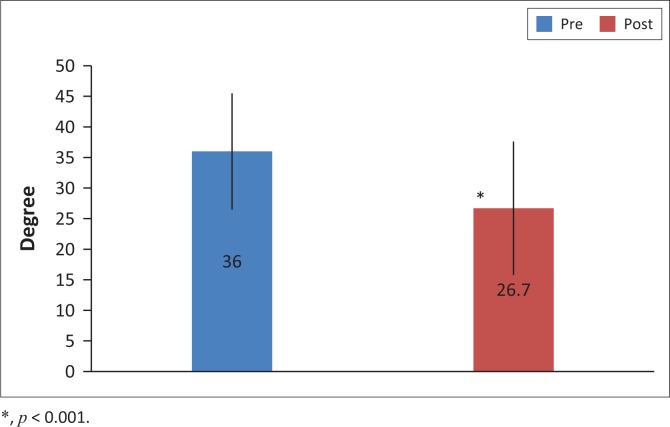
Mean knee extension range of motion (in degrees) ± standard deviation pre- and post-intervention in the group that received neurodynamic technique following passive stretching.

**FIGURE 5:**
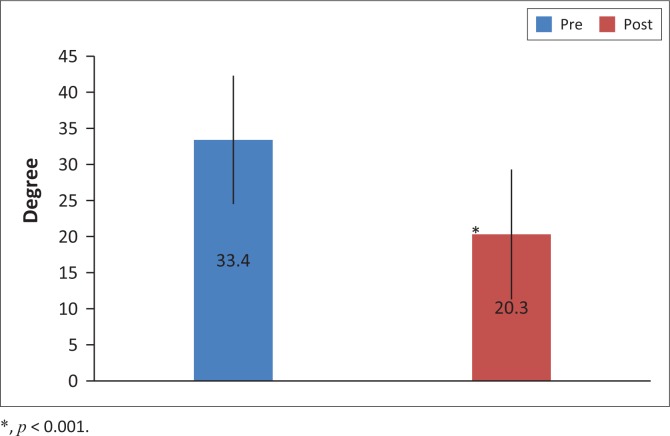
Mean knee extension range of motion (in degrees) ± standard deviation pre- and post-intervention in the group that received quadriceps activation following passive stretching.

There was a significant improvement in hamstring flexibility in the group that received QA following PS compared to the group who received PS only (13.4 ± 12.1° vs. 6.2 ± 6.4°, *p* < 0.05,95% confidence interval (CI): 0.1–13.4°; Figure 2). There was a significant improvement in hamstring flexibility post-intervention in PS compared to pre-intervention where the knee extension increased on average by 6.2 ± 6.4° (30.5 ± 10.8 vs. 36.6 ± 9.5°, 95% CI: 29.6–37.5°, *p* < 0.001; Figure 3). There was a significant improvement in hamstring flexibility post-intervention in the ND group compared to pre-intervention where the knee extension increased by 9.3 ± 6.2° (26.7 ± 10.9° vs. 36.0 ± 9.5°, 95% CI: 27.4–35.3°, *p* < 0.001; Figure 4). There was a significant improvement in hamstring flexibility post-intervention in the QA group compared to pre-intervention where the knee extension increased by 13.4 ± 12.1° (20.3 ± 9.0° vs. 33.4 ± 8.9°, 95% CI: 22.8–30.7°, *p* < 0.001; Figure 5). Cohen’s effect indicated the presence of a moderate effect size for ND compared to PS (Cohen’s *d* = 0.5).

## Discussion

The group that received quadriceps activation following passive hamstring stretching improved significantly compared to the group that received passive stretching only. This could be because of the reciprocal inhibition effect (Crone 1993). Reciprocal inhibition occurs through a spinal cord primitive reflex arc when the antagonist muscle relaxes in response to the activation of the agonist muscle. Therefore, the group that had the quadriceps activation had more inhibition to the antagonist muscle (hamstring) compared to the group that only received passive stretching.

Nishikawa et al. (2015) studied the immediate effect of passive stretching compared to active stretching on hamstring muscle flexibility. They found that passive stretching was more effective than active stretching in increasing hamstring muscle flexibility (Nishikawa et al. 2015). This is contrary to the findings of our study; however, they did not precede active stretching with passive stretching as was done in our study. Passive stretching prior to active stretching could prepare the muscle for reciprocal inhibition and result in better outcomes.

There was no significant difference in hamstring muscle flexibility between the ND group and PS group. This could be a result of the fact that participants received two sets of 10 repetitions of the neurodynamic technique, which may have not been sufficient to create a statistical difference between groups. Therefore, a future study is recommended to include three sets of 10 repetitions of the neurodynamic technique to determine the effect on hamstring flexibility. Even though the difference between the ND group and PS group was not statistically significant, it was clinically important. Cohen’s effect indicated the presence of a moderate effect size for the neurodynamic technique compared to passive stretch (Cohen’s *d* = 0.5). This supports the idea of having more repetitions of the neurodynamic technique in order to have statistically significant differences between groups.

Bandy and Irion (1994) and Cipriani et al. (2012) found that 30 seconds of passive stretch is as effective as 60 seconds of passive stretch (Bandy & Irion 1994; Cipriani et al. 2012), supporting the findings of our study. In our study, the group who received 30 seconds passive stretch had significant improvements in hamstring flexibility post-intervention compared to pre-intervention. Similarly Johnson et al. (2014) support our findings, as they found a significant improvement in hamstring muscle flexibility following passive stretching (Johnson et al. 2014).

Our findings may be helpful in physiotherapy clinical settings where there is a need to improve hamstring flexibility. Physiotherapists can combine passive stretching with the quadriceps activation technique in order to achieve the optimal effect on hamstring flexibility in patients who have tight hamstrings. This may help in reducing the risk of having postural changes that could result in musculoskeletal pain and dysfunction (Borman et al. 2011; Jozwiak et al. 1997; Radwan et al. 2014; Sadler et al. 2017).

The limitations of this study include the small sample size, unequal repetitions of neurodynamic and quadriceps activation techniques and a lack of carry-over measurements. Future studies are recommended to find the effect and carry-over of repetitive use of neurodynamic and quadriceps activation techniques on hamstring muscle flexibility with a bigger sample size and more repetitions of the neurodynamic technique.

## Conclusion

Quadriceps muscle activation following passive stretching of hamstring muscle is an effective way to improve hamstring flexibility.

### Clinical implications

Quadriceps activation following passive hamstring stretching can be used in physiotherapy settings to improve hamstring flexibility in people who have tight hamstrings, resulting in postural changes or musculoskeletal pain.
